# Combined Adiponectin Deficiency and Resistance in Obese Patients: Can It Solve Part of the Puzzle in Nonalcoholic Steatohepatitis

**DOI:** 10.3889/oamjms.2015.057

**Published:** 2015-05-30

**Authors:** Ahmed Salman, Mona Hegazy, Soheir AbdElfadl

**Affiliations:** *Faculty of Medicine, Cairo University, Internal Medicine, Cairo, Egypt*

**Keywords:** Combined adiponectin deficiency and resistance in obese patients, can it solve part of the puzzle in NASH

## Abstract

**BACKGROUND:**

Nonalcoholic fatty liver disease (NAFLD) has become the most prevalent cause of liver disease, nonalcoholic steatohepatitis (NASH) and fibrosis in obese patients identifies the risk group with increased incidence of liver-related deaths.

**AIM:**

To clarify the role of serum adiponectin and its receptor liver gene expression in the progression of liver damage in NAFLD.

**METHODS:**

Fifty four (54) obese patients with NAFLD preliminary diagnosed by liver ultra-sound were recruited. Full medical history, anthropometric measurement, biochemical studies, serum adiponectin level, liver biopsy for histological examination and NAS score to identify NASH patients, and assessment of adiponectin receptor gene expression by RT-PCR, were conducted for each patients. Fifteen ages matched average weight healthy adult had been chosen as a control for serum adiponectin level.

**RESULTS:**

According to NAS score, patients were divided into non- NASH (8 patients), and NASH (46 patients). Serum adiponectin level was significantly lower in NAFLD patients compared to normal participants (p < 0.004). Serum adiponectin level was lower in NASH patients (4.437 ± 2.569 ng/dl in NASH vs. 5.138 ± 2.841 ng/dl in non-NASH). Adiponectin receptor liver gene expression was lower in NASH patients (0.8459 ± 0.4671 vs. 1.0688 ± 0.3965 in non-NASH).

**CONCLUSION:**

Both adiponectin deficiency and resistance had a role in progression of simple liver steatosis to severe injury in obese patients.

## Introduction

Nonalcoholic fatty liver disease (NAFLD) is closely associated with obesity and insulin resistance, and is now recognized to represent the hepatic manifestation of the metabolic syndrome. Since the term nonalcoholic steatohepatitis (NASH) was first coined by Ludwig et al. in 1980, [1] the prevalence of NAFLD has risen rapidly in parallel with the dramatic rise in population levels of obesity and diabetes, [2] resulting in NAFLD, now representing the most common cause of liver disease in the western world [3].

Among morbidly obese patients undergoing bariatric surgery, the prevalence of simple fatty liver is 47%, the prevalence of NASH is 27-42%, and cirrhosis occurs in 2.9-3.9% [4]. Whilst simple steatosis is thought to be a relatively benign entity, NASH has the potential to lead to morbidity and mortality.

Adipotectin is a protein which encoded by the *ADIPO Q* gene [5], this gene localized to chromosome 3q27, a region highlighted as affecting genetic susceptibility to type 2 diabetes and obesity [6]. Acute injection of recombinant adiponectin enriched with the high molecular weight (HMW) oligomers results in a marked activation of AMP activated kinase (AMPK) in the liver, while chronic infusion with this protein leads to prolonged alleviation of hyperglycemia and insulin resistance [7].

Tow adiponectin receptors (Adipo R), have been identified, Adipo R1 is abundantly expressed in skeletal muscles, whereas adipo R2 is present predominantly in the liver [8].

Adenovirus-mediated expression of Adipo R1 or Adipo R2 in the liver of mice significantly increased fatty acid oxidation, and tend to decrease hepatic triglycerides content [9]. Obesity decrease plasma adiponectin level and Adipo R1/R2 expression, causing adiponectin resistance [10].

Mice without adiponectin show an increased lipid accumulation under normal feeding; this pre-existing hepatic steatotic condition might be the direct consequence of dysregulated mitochondrial respiratory chain [11]. Adiponectin treatment restores the mitochondrial respiratory chain (MRC) activities, decrease the level of mitochondrial lipid peroxidation products through regulating hepatic mitochondrial functions [9].

## Patients and Methods

### Patients

The current study is a prospective study, performed in Kasr Al-eini hospital, Internal Medicine Out-patient Clinic (Liver and Gastroenterology Clinic), Faculty of Medicine, Cairo University, over a 6 months period (June-November 2011).

NAFLD patients’ preliminary diagnosed by liver ultrasound scan showing a picture of fatty liver ± elevated liver enzymes.

The selection of participants in this study was based on the following inclusion criteria; males and females, aged between 18 and 60 years, with Body Mass index (BMI) over 30 kg/m^2^, having a bright liver on liver ultrasound scan (picture suggestive of hepatic steatosis), and no history of alcohol intake. While participants with hepatitis C or B infection, patients with known causes of liver disease (autoimmune, genetic, or drug induced), patients with major systemic conditions and pregnant women were excluded from the study.

Fifty four male and female participants (50 females and 4 males) had a complete work-up including, a detailed medical history, general physical examination, anthropometric measurements, serum biochemistry profiles; including serum adiponectin, hepatitis markers (HCV antibody, and HBVs antigen), liver ultrasound scan, and True-cut ultrasound guided liver biopsy.

Fifteen healthy ages matched average weight (BMI < 25 Kg/m^2^) participants, with normal appearance of the liver during scanning, and normal liver enzymes, had been chosen as a control for serum adiponectin level.

The study protocol conformed to the ethical guidelines of the 1975 Declaration of Helsinki, and was approved by Cairo University Hospital Research Ethics Committee (REC) in 28-5-2011 (N-7-2011).

Written informed consents were obtained from all participants in the study.

### Methods

All participants were interviewed for their medical history, and information on life style factors. The weight and height of each participant were measured while the participant was clothed only in a light gown, and the body mass index was calculated as body weight divided on height squared (Kg/m^2^), obesity defined as a BMI ≥ 30 Kg/m^2^. The waist circumference was measured midway between the lowest rib margin and the iliac crest in a standing position, by the same examiner.

Laboratory investigations, included serum alanine aminotransferase (ALT), aspartate aminotransferase (AST), γ-glutamyl transpeptidase (GGT), alkaline phosphatase (ALP), total and conjugated bilirubin, prothrombin concentration, fasting blood sugar, and lipid profile (total cholesterol, low density lipoprotein (LDL) cholesterol, high density lipoprotein (HDL) cholesterol, and triglycerides). Serum Adiponectin level was measured using the quantitative Sandwish enzyme immunoassay technique. A monoclonal antibody specific for the adiponectin or resistin globular domain were used.

Liver ultrasound scan (US) performed by the same operator (MH), using a Toshiba Aplio xv scanner equipped with a broad band 2.5-5 MHZ curved-array probe to assess the presence of liver steatosis (“bright liver”), which was defined and graded as: 1- a diffuse hyper echoic echo texture (bright liver); 2- increased liver echo texture compared with the kidney; 3- vascular blurring; and 4- deep attenuation [12]. Steatosis was graded using this semi-quantitative scale from 1 to 4.

All participants were eligible for liver biopsy, based on the presence of hepatic steatosis in liver US ± elevated liver enzymes, and normal prothrombin concentration. All the samples were evaluated by the same pathologist (AM). They were fixed in ten percent neutral buffered formalin, embedded in paraffin blocks, then cut into five micrometer thick sections and stained with hematoxylin & eosin. They were examined under the light microscope for histo-pathological evaluation.

In a subsequent analysis of liver biopsies, liver damage was assessed by means of the NAFLD activity score (NAS), which is the sum of steatosis (scale from 0-3), lobular inflammation (scale from 0-3), and hepatocellular ballooning (scale from 0-2), according to Kleiner et al, 2005 [13]. This scoring system addresses the full spectrum of lesions of NAFLD, and allows a diagnostic categorization into NASH, border-line NASH, or non- NASH. Fibrosis staging (evaluated separately from NAS) from 0-4 scales (where 0= no fibrosis, and 4= cirrhosis) according to the classification set by Brunt, et al in 1999 [14].

Our participants were divided into two groups according to NAS score; NASH positive patients (46); (24 border-line NASH, and 22 NASH “2 of them had fibrosis”), and another group had no NASH (8 patients).

Quantitative Real time-polymerase chain reaction (RT-qPCR) was used for assessment of adiponectin receptor gene expressions in liver tissue. [15]. Total RNA was extracted from liver tissue using SV total RNA isolation system (Promega, Madison, WI, USA), RNA purification, Reverse Transcription into cDNA using RT-PCR kit (Stratagene, USA), then the real-time PCR result was analyzed with the step one applied biosystem software.

### Statistical Methods

Data were statistically described in terms of mean ± standard deviation (± SD), and frequencies (number of cases) and relative frequencies (percentages) when appropriate. Comparison of numerical variables between the study groups was done using Kruskal Wallis test with multiple groups’ comparisons. For comparing categorical data, Chi square (χ2) test was performed. Exact test was used instead when the expected frequency is less than 5. A probability value (p value) less than 0.05 was considered statistically significant. All statistical calculations were done using computer programs SPSS (Statistical Package for the Social Science; SPSS Inc., Chicago, IL, USA) version 15 for Microsoft Windows.

## Results

The present study included 54 patients (50 femals 42.6%, and 4 males 7.4%), aged 43.17 ± 7.3 years, have NAFLD suspected by liver ultrasound scan and confirmed by liver biopsy. 15 age-matched average weight (BMI < 24.9 kg/m^2^) healthy participants were the control group.

Anthropometric measurements and laboratory studies of NAFLD patients are shown in Table 1.

**Table 1:** Clinical and laboratory data of all NAFLD patients

Parameter	Mean ± SD
BMI (kg/m²)	34.77 ± 3.761
Waist (cm)	106.13 ± 14.993
ALT (IU/L)	33.24 ± 19.061
AST (IU/L)	33.02 ± 20.141
GGT (IU/L)	47.56 ± 35.499
FBS (mg/dl)	92.85 ± 9.472
Total-C (mg/dl)	206.15 ± 33.093
LDL-c (mg/dl)	104.19 ± 21.267
HDL-c (mg/dl)	48.04 ± 14.403
Triglycerides (mg/dl)	171.56 ± 43.824

ALT; alanine aminotransferase, AST; aspertate aminotransferase, BMI; body mass index, FBS; fasting blood sugar, GGT; gamma glutemyl transpeptidase, HDL-c; high density lipoprotein cholesterol, LDL-c; low density lipoprotein cholesterol, Total-C; total cholesterol.

According to NAS score in liver biopsy of NAFLD patients; 46 patients (44 females, and 2 males) proven to had NASH and border line NASH, and only 8 patients (6 females, and 2 males) were non-NASH.

Clinical and laboratory data of NASH and non- NASH patients are shown in Table 2.

**Table 2:** Clinical and laboratory data of NASH and non-NASH patients

Parameter	NASH		P value
	Yes n.46	No n.8	
Age (years)	42.71 ± 4.52	45.75 ± 6.27	0.29
BMI (kg/m²)	34.98 ± 3.95	33.59 ± 2.19	0.643
Waist (cm)	106.65 ± 15.53	103.13 ± 11.78	0.487
ALT (IU/L)	35.78 ± 19.36	18.63 ± 7.43	0.005*
AST (IU/L)	34.41 ± 21.41	25.0 ± 6.16	0.223
GGT (IU/L)	51.63 ± 36.58	24.13 ± 14.62	0.006*
FBS (mg/dl)	120.72 ± 30.58	121.63 ± 23.97	0.697
T.cholesterol (mg/dl)	203.0 ± 31.53	224.25 ± 38.22	0.161
LDL-c (mg/dl)	123.15 ± 18.67	110.13 ± 33.72	0.512
HDL-c (mg/dl)	47.41 ± 14.95	51.63 ± 10.82	0.176
Triglycerides (mg/dl)	173.52 ± 43.95	160 ± 44.21	0.465

N; number of patients in this group

* P

significant less than0.05.

NAFLD patients either NASH or non-NASH almost was matched in age (NASH 42.71± 4.52 years, non-NASH 45.75 ± 6.27 years, p = 0.29).

BMI, and waist circumference were slightly higher in NASH patients but the differences were not significant. Adiponectin level among the obese patients with NAFLD was 4.54 ± 2.5454 ng/L while it was significantly higher in normal weight healthy participants; 6.434 ± 0.796 ng/L (p = 0.004).

Adiponectin levels and its gene expression in liver biopsy in NASH and non-NASH patients are shown in Table 3.

**Table 3:** Serum Adiponectin level and its liver gene expression in NASH and non-NASH patients

Parameter	NASH		P value
	Yes N = 46	No N = 8	
Adiponectin serum level (ng/L)	4.437 ± 2.569	5.138 ± 2.841	0.519
Adiponectin receptor liver gene expression	0.8459 ± 0.4671	1.0688 ± 0.3965	0.147

Correlating serum adiponectin level with all the clinical and laboratory parameters we assessed including liver enzymes, shown no significant correlation except for the triglycerides level which was negatively correlated with serum adiponectin (r -0.61, p < 0.0001).

## Discussion

NAFLD represents a spectrum of disorders characterized by predominantly macro-vesicular hepatic steatosis that occurs in individuals in the absence of consumption of alcohol in amounts considered harmful to the liver [16]. NAFLD is a leading cause of chronic liver disease and its incidence is raising worldwide [17].

Measuring serum adiponectin “which is a protein hormone secreted by adipocytes” may serve as a predictor of progressive liver pathology in NAFLD. Adiponectin is an insulin sensitizing hormone that has multiple beneficial effects on obesity-related medical complications [18]. It was found that serum adiponectin level significantly increased after weight reduction [19].

In the current study, adiponectin serum level was significantly lower in NAFLD patients than in normal participants (P Value = 0.004); and also its level was lower in NASH patients in comparison to patients with simple steatosis.

Several previous studies showed that serum adiponectin level was lower in NASH patients than non-NASH patients. Also, Baranova and colleagues in 2006; stated that, serum adiponectin level was the only predictor of NASH [20-22].

In contrast to this, Savvidou and colleagues in 2009 founded that serum adiponectin concentration was not associated with NAS Score [23].

AdipoR1 is abundantly expressed in skeletal muscles, whereas adipoR2 is present predominantly in the liver [8]. Expression of Adipo R2 significantly increased the expression of gene encoding molecules involved in glucose uptake such as glucokinase (GCK), [27] which appeared to be one possible mechanism by which Adipo R2 expression in the liver apparently improved diabetes.

The expression of adiponectin receptors is correlated with insulin levels. It has been found that their levels are reduced in mouse models of diabetics, particularly in skeletal muscle and adipose tissue [26]. Also Adipo R1 or Adipo R2 in the liver of mice significantly increased fatty-acid oxidation, and tended to decrease hepatic triglyceride content [9].

Recently Adamska et al, 2012 showed that, down regulation of Adipo R1 and Adipo R2 in obesity plays a causal role at least in the development of insulin resistance [5].

As a result of this, obesity decreases not only plasma adiponectin levels but also Adipo R1/R2 expression, thereby causing adiponectin resistance and leading to insulin resistance, which in turn aggravates hyperinsulinaemia forming a ‘vicious cycle’ [10].

It has been found that lipid load in adipose tissue of obese patients is a source of a pro-inflammatory systemic environment mechanistically implicated in obesity and diet induced insulin resistance. This is pro-inflammatory state is characterized among others by increased TNF and leptin level with a decrease in adiponectin release [28].

Regarding our study, we found that adiponectin receptor gene expression in liver biopsy was lower in NASH patients in comparison to non-NASH patients.

In parallel with our results, two previous studies founded that adiponectin receptor gene expression in liver biopsy was lower in NAFLD patients, especially in the NASH group [24, 25].

The result of the current study showed that; not only the adiponectin serum level was low in patients with NAFLD, but also its level decreased more in the presence of NASH rather than simple steatosis, and even more the Adipo R gene expression in livers of patients with NASH showed a lower level than patients with simple steatosis.

Adipo R2 receptor depletion indicates that, not only adiponectin serum level depletion had a role in progression of severity of NAFLD, but also adiponectin resistance may help in exploring part of the missing puzzle in the pathogenesis of NAFLD.

We should point out some limitations of the study, first; the relatively limited number of patients we worked on with unequal distribution of sex in the study group, second; the unequal distribution of NASH, and non-NASH cases, and finally; liver biopsies were studied by one pathologist. Our excuse to all these limitations that, most of the NAFLD Egyptian patients believe that fatty liver is a common benign condition, so they refused the idea of liver biopsy.

In conclusion, both adiponectin deficiency and resistance had a role in progression of simple liver steatosis to severe injury in obese Egyptian patients.
